# Group and Basis Restricted Non-Negative Matrix Factorization and Random Forest for Molecular Histotype Classification and Raman Biomarker Monitoring in Breast Cancer

**DOI:** 10.1177/00037028211035398

**Published:** 2021-08-06

**Authors:** Xinchen Deng, Kirsty Milligan, Ramie Ali-Adeeb, Phillip Shreeves, Alexandre Brolo, Julian J. Lum, Jeffrey L. Andrews, Andrew Jirasek

**Affiliations:** 1 Department of Physics, The University of British Columbia Kelowna, Canada; 2 Department of Statistics, The University of British Columbia, Kelowna, Canada; 3 Department of Chemistry, University of Victoria, Victoria, Canada; 4 Department of Biochemistry and Microbiology, University of Victoria, Victoria, Canada; 5 Trev and Joyce Deeley Research Centre, BC Cancer, Victoria, Canada

**Keywords:** Raman spectroscopy, radiation response monitoring, random forest

## Abstract

Raman spectroscopy is a non-invasive optical technique that can be used to investigate biochemical information embedded in cells and tissues exposed to ionizing radiation used in cancer therapy. Raman spectroscopy could potentially be incorporated in personalized radiation treatment design as a tool to monitor radiation response in at the metabolic level. However, tracking biochemical dynamics remains challenging for Raman spectroscopy. Here we developed a novel analytical framework by combining group and basis restricted non-negative matrix factorization and random forest (GBR-NMF-RF). This framework can monitor radiation response profiles in different molecular histotypes and biochemical dynamics in irradiated breast cancer cells. Five subtypes of; human breast cancer (MCF-7, BT-474, MDA-MB-230, and SK-BR-3) and normal cells derived from human breast tissue (MCF10A) which had been exposed to ionizing radiation were tested in this framework. Reference Raman spectra of 20 biochemicals were collected and used as the constrained Raman biomarkers in the GBR-NMF-RF framework. We obtained scores for individual biochemicals corresponding to the contribution of each Raman reference spectrum to each spectrum obtained from the five cell types. A random forest classifier was then fitted to the chemical scores for performing molecular histotype classifications (HER2, PR, ER, Ki67, and cancer versus non-cancer) and assessing the importance of the Raman biochemical basis spectra for each classification test. Overall, the GBR-NMF-RF framework yields classification results with high accuracy (>97%), high sensitivity (>97%), and high specificity (>97%). Variable importance calculated in the random forest model indicated high contributions from glycogen and lipids (cholesterol, phosphatidylserine, and stearic acid) in molecular histotype classifications.

## Graphical abstract

**Graphical Abstract:**
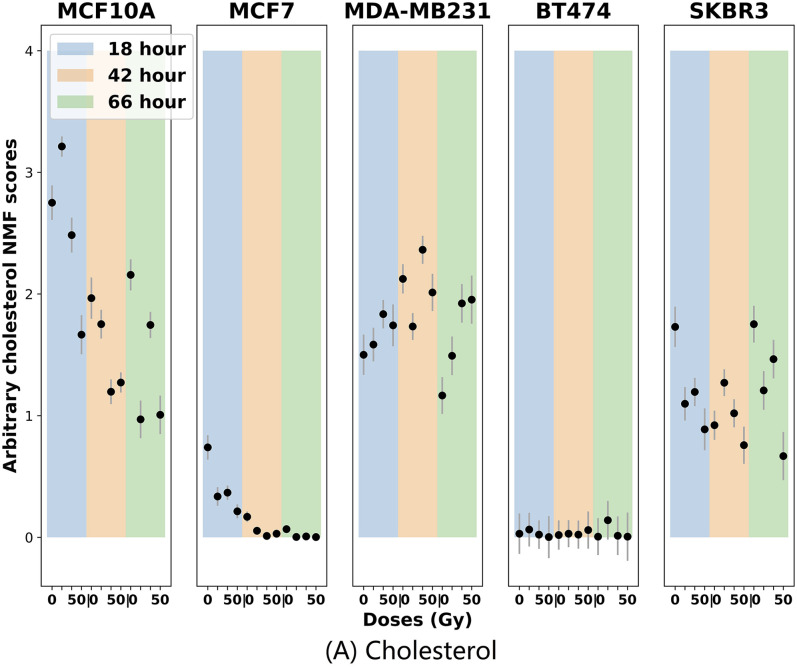

## Introduction

Radiation therapy (RT) is a treatment prescribed to approximately 50% of cancer patients.^1^ Currently, RT treatment plans are often designed and evaluated based on morphological information acquired by imaging modalities such as computed tomography (CT) and magnetic resonance imaging (MRI).^2–4^ RT treatment schemes do not incorporate information on biochemical dynamics in tumours as part of the treatment design and evaluation. However, tumor cells possess signaling pathways and metabolic processes inherently distinct from normal cells, leading to non-uniform cellular radiation response.^5^ For example, breast cancer as a heterogeneous disease can be classified into molecular subtypes based on the immunochemical molecular histotypes.^6^ Histopathological assessment based on the expression of estrogen receptor (ER), progesterone receptor (PR) and the human epidermal growth factor receptor 2 (HER2) has been included in breast cancer classification system.^7–9^ A strong interest in designing personalized treatments based on the molecular subtypes has been advocated.^10,11^ Although molecular subtypes and histotypes are often regarded as essential when making treatment decisions, the biomolecular dynamics for each subtype and histotype remain unclear and challenging to track in response to treatment. Developing a tool to identify and monitor biomarkers that can be used as targets in cancer treatment (e.g., to develop radiosensitizers) is necessary.

Raman spectroscopy (RS) is a noninvasive optical method applicable to soft tissues and cells to monitor and evaluate biological changes in response to stimuli on a cellular level in the tumor microenvironment. RS provides information on multiple biomarkers in cell samples using the vibrational fingerprint of the biochemicals. Previous works have demonstrated that RS can detect radiation-induced biochemical variations in cancer cells and tumours.^12–17^Performing unsupervised dimension reduction (e.g., principal component analysis (PCA) and non-negative matrix factorization (NMF)) helps overcome the complexity of RS data analysis. Applying unsupervised dimension reduction techniques has helped identify glycogen and lipids as primary biochemicals responsible for spectral changes on cellular and tumour samples exposed to ionizing radiation.^12–18^ However, the primary difficulty encountered by the commonly used unsupervised dimension reduction algorithms is interpreting the decomposed components or loading vectors. This limitation often results in unidentifiable bases which can be related to specific chemicals.

Negative matrix factorization is an unsupervised learning technique used for dimensionality reduction.^19^ Due to the non-negativity assumption, NMF can improve the interpretability of decomposed components in spectroscopic data over other dimension reduction techniques.^16,20,21^ NMF, as an unsupervised analytical method, also has its limitations. For example, matching chemical bases decomposed from unsupervised NMF to the actual biochemicals present within a Raman spectrum is a complex task. To address this limitation, a group and basis restricted non-negative matrix factorization (GBR-NMF) algorithm was used in the current study to conduct dimensionality reduction.^22^ GBR-NMF constrains biochemicals during dimension reduction and allows for better evaluation of chemicals of interest.^23^

In the present study, GBR-NMF is combined with a random forest (RF) classifier to form a data analytical framework. RF is an ensemble classifier that generates the forest’s classification result based on the voting results of a collection of decision tree classifiers within the forest.^24^ After GBR-NMF decomposes the Raman spectra, a random forest classifier was applied to the GBR-NMF decomposed scores of constrained chemicals. RF can classify the chemical scores into cell lines and molecular histotypes while providing information on the relative contribution of individual biochemicals in the classification via variable importance.

The current work aims to demonstrate that the GBR-NMF and random forest combined (GBR-NMF-RF) data analytical framework can accurately classify molecular histotypes of breast cell line RS data acquired from cells exposed to clinically relevant radiation doses. This data analytical framework also has the advantage of revealing the high-contributing chemicals in the classification. The combination of RS and GBR-NMF-RF provides a robust technique to track the biochemical dynamics across breast cancer molecular histotypes throughout treatment. The data used to test the model are Raman spectra acquired on one normal breast cell line, MCF10A, and four breast cancer cell lines (MCF-7, BT-474, MDA-MB-231, and SK-BR-3) irradiated with single doses of radiation (0, 10, 30, 50 Gy). These cell lines span a range of molecular histotypes and are presented in Table I. The GBR-NMF was performed on RS cellular data with two reference libraries of basis biochemicals (containing 16 and 20 biochemical bases, respectively) for comparison. RF was applied to the decomposed biochemical scores to classify the breast cell lines into different molecular histotypes, yielding classification results with high accuracy (>97%), high sensitivity (>97%), and high specificity (>97%). The variable importance ranking reveals high contributions from the Raman spectra of the biomarkers glycogen and lipids (cholesterol, phosphatidylserine, and stearic acid) in molecular histotype classifications.

**Table I.** Immunohistochemical profiles of the breast cell lines.

Cell lines	HER2	PR	ER	Ki67	Subtype	Cancer
MCF10A	–	–	–	Low	Basal	No
MCF-7	–	+	+	Low	Luminal A	Yes
MDA-MB-231	–	–	–	High	Basal-like	Yes
BT-474	+	+	+	High	Luminal B	Yes
SK-BR-3	+	–	–	High	HER2	Yes

## Materials and Methods

### Breast Cancer Cell Line Molecular Histotypes

The molecular histotypes of the breast cell lines are summarized from previous studies.^12,25,26^ The previously established immunohistochemical information is shown in Table I. The estrogen receptor (ER), progesterone receptor (PR), human epidermal growth factor receptor-2 (HER2), cell proliferation marker Ki-67 histotype statuses are determined by immunohistochemistry tests.^27,28^

Based on the immunohistochemistry of the histotypes, the cancer cell lines selected can be categorized into subtypes, such as Luminal A (with ER and/or PR+, HER2−, low Ki67), Luminal B (HER2+) (with ER and/or PR+, HER2+), HER2 (with ER−, PR−, HER2+) and Basal-like (with ER−, PR−, HER2−, low Ki67).^8,9^

### Raman Spectroscopy Reference Biochemical Library Selection

We constructed two chemical basis libraries to cover a range of biochemicals known to be present within the Raman cellular spectra, which includes carbohydrates, lipids, enzymes, amino acids, and nucleic acids.^29–32^ The chemical reference libraries were built based on the biological composition of cells and biomolecular dynamics in cancer metabolism. Building a model is a trade-off between complexity and the ability to cover a complete set of molecular compounds. Hence, we reported both simpler (16-chemical) and more complex (20-chemical) libraries to assess the trade-off in complexity and model performance.

A 16-chemical library is presented in Table S1 (Supplemental Material). Glycogen, glucose, and lipids are included in the reference library since previous studies have traced them as Raman biomarkers for radiation treatment response.^13–16,23,33^ A wide range of lipids were selected, including; cholesterol, glycerol, glyceryl tripalmitoleate, oleic acid, palmitic acid, phosphatidylcholine, phosphatidylserine, and stearic acid. The RS spectra of lipids are highly alike,^30–32^ and previous studies could not distinguish the contribution of individual lipids in radiation response.^12,16^ Citric acid and lactic acid were added to the library, given they are downstream products of glucose metabolism in cancer metabolic pathways. After glucose is consumed and changes to pyruvate via glycolysis, pyruvate will feed into the citric acid cycle, also known as the tricarboxylic acid cycle (TCA cycle), to make energy or be converted to lactic acid.^34,35^ Coenzyme A (CoA) was included in the library as it is involved in the oxidative decarboxylation of pyruvate into acetyl-CoA, which has a vital role in fatty acid metabolism.^36^ DNA is taken into account as an essential chemical to monitor since RT delivers ionizing radiation to the tumor, leading to DNA damage, particularly DNA double-strand breaks.^37^ This library also includes glutathione and mannose. Glutathione is the most abundant antioxidant and non-protein thiol present in mammalian tissues due to cellular redox homeostasis (the maintenance of many cellular processes including signalings, etc.), which can implicate tumour initiation, progression, and treatment response.^38,39^ Mannose is a sugar monomer carbohydrate participating in glycosylation (the reaction in which a carbohydrate is attached to a hydroxyl or other functional group).^40^ Abnormal growth factor signalling in cancer development can be attributed to specific types of glycosylation.^41^

A 20-chemical extended library was also constructed to exploit GBR-NMF’s ability to constrain chemical bases of interest (Table S2). For example, amino acids or proteins were difficult to trace in previous studies by unsupervised dimension reduction methods,^12–16,33^ although they have significant roles in cancer metabolism. Two amino acids of interest (valine and leucine) were selected for inclusion in the 20-basis library due to their importance in metabolic pathways related to amino acids in breast cancer.^42^ They are branched-chain amino acids that serve as alternative sources of organic molecules that can also serve as indirect substrates for the TCA cycle.^43^ Collagen is the major fibrous protein in the extracellular matrix and connective tissue.^44^ However, given the Raman spectral data used in the current study are cellular, it is only included in the 20-chemical extended library to test the effect on the model. A triglyceride mixture was also added to the extended library to test whether the analytical methods could distinguish chemicals with similar spectra, such as triglycerides and glyceryl tripalmitoleate. The triglyceride mixture contains glyceryl tridecanoate, glyceryl tridodecanoate, glyceryl trimyristate, glyceryl trioctanoate, and tripalmitin, which all have similar RS spectra.^32^ The RS spectrum of glyceryl tripalmitoleate resembles the triglyceride mixture spectrum with minor differences at 1266 and 1656 cm^−1^.^32^

All chemicals other than the triglycerides mixture were supplied by Sigma Aldrich, St. Louis, MO, USA. The triglycerides mixture is a certified reference material of lipid standards purchased from Supelco (Bellefonte, USA).

### Cell Cultures

All cell samples were cultured as described previously by Meksiarun et al.^12^ Four types of human epithelial breast tumour cells (MCF-7, BT-474, MDA-MB-231, and SK-BR-3) were cultured in DMEM medium supplemented with 10% fetal bovine serum (FBS, Hyclone). MCF10A, the normal breast cells were cultured in MEBM medium supplemented with MEGM SingleQuots (Lonza, Basel, Switzerland). Five percent CO_2_ was supplied to the cells in an incubator, and the temperature was maintained at 37.8 ℃. The cells were harvested and plated into 12 identical flasks as equivalent aliquots of cell suspension. The flask cultures were incubated for 96 h to reach approximately 50% confluency. Fresh media were replaced in the cultures 1 h before irradiation. All flasks were removed from the incubator for less than 40 min before irradiation.

### Cell Irradiation and Harvesting

For irradiation, flasks were placed between two 5-cm-thick pieces of water equivalent plastic solid water (Gammex RMI, USA) in order to provide electron buildup at the layer of the cells.^12^ Irradiation was delivered using a 6 MV photon beam from a Varian 21EX linear accelerator (Varian Medical Systems Inc., USA) with a dose rate of 6 Gy min^–1^ at the isocenter with single fractions of 0, 10, 30, or 50 Gy. Each dose was delivered to three flasks.

At 18, 42, and 66 h post-irradiation, one flask from each dose was harvested for RS acquisition. The harvested cells were washed with phosphate-buffered saline (PBS, Hyclone) and then re-suspended in PBS plus 10% FBS. The suspension was centrifuged into a pellet (without isolating cells) and placed onto a 5 mm-thick MgF_2_ disk (Janos Technology Inc., USA) based on a previously established protocol.^14^

### Raman Spectroscopy Acquisition (Breast Cells and Reference Biochemicals) and Pre-Processing of Spectra

The Raman spectra of cells were acquired based on a previously developed protocol.^33,12–14^ After the cell pellet was placed onto a 5 mm-thick MgF_2_ disk, cells were user-identified and chosen for ease of Raman sampling (not overly splayed out, good sampling volume). Raman spectra of cells were acquired with an inVia Raman microscope (Renishaw Inc., USA) with a 100× dry objective (NA = 0.9) (Leica Microsystems, Germany), a 600 lines per mm diffraction grating, a thermoelectrically cooled iDus charge-coupled device (CCD) detector (Andor Technology, UK). The acquisition parameters were 10 s acquisition time per cell and a 425–1820 cm ^− 1^ spectral window. A 785 nm laser (Renishaw-based diode laser) was used for excitation, providing a laser power density at the sample of ∼0.5 mW/μm^−3^. The size of the sampling volume was ∼2×5×10 μm (aligned with the centre of the selected cell), allowing a single acquisition to represent the spectrum of a single cell ( ∼10 μm diameter). The laser focal spot on the Renishaw system is rectangular in shape due to the nature of the laser assembly (Renishaw-based 785 nm diode laser). Twenty spectra from single cells were collected from each cell line, resulting in 240 Raman spectra acquired over four doses (0, 10, 30, and 50 Gy) and three harvesting times (18, 42, and 66 h), which accumulated in a total of 1200 acquired spectra.

Spectra of chemicals presented in Tables S1 and S2 (liquid or solid forms) were acquired using a Renishaw inVia Raman microscope (Renishaw Inc.) with a 100× dry objective (NA = 0.9) (Leica Microsystems, Germany), a 1200 lines/mm diffraction grating, a 10-s exposure time, and a 785 nm laser (Renishaw). The spectra of all 20 reference chemicals are presented in Fig. S1. The reference chemical basis spectra were acquired at a higher spectral resolution in order to distinguish any overlapping features. However, all spectra were interpolated onto the same wavenumber axis (resolution) prior to performing our analysis. No adverse effects of the interpolation or higher spectral resolution of the basis spectra were observed in the analysis.

Each cell spectrum was processed with a cosmic ray removal program from WiRE (Renishaw Inc.). All the reference chemical and cosmic-ray-removed cellular spectra were processed with baseline subtraction (estimate and subtract the baseline due to the substrate and biological fluorescence) and normalization under the curve (equal to 1) in Matlab (The Mathworks, Inc.).

### Data Analysis: GBR-NMF-RF Data Analytical Framework

#### Non-Negative Matrix Factorization and Group and Basis Restricted Non-Negative Matrix Factorization

NMF decomposes the non-negative data matrix $X\in {\mathbb{R}}_{\geq 0}^{n\times p}$ (where *n* is the number of samples and *p* is the number of variables in the data matrix) into two lower rank non-negative matrices $W\in {\mathbb{R}}_{\geq 0}^{n\times q}$ and $H\in {\mathbb{R}}_{\geq 0}^{q\times p}$ such that

X≈WH<label>(1)</label>

where *q* is the number of underlying factors. This is generalized from the true decomposition of *X*

X=WH+ɛ<label>(2)</label>

with $ɛ\in {\mathbb{R}}_{\geq 0}^{n\times p}$ representing the residual error.^19,22^

Based on the NMF, group and basis restricted non-negative matrix factorization (GBR-NMF) variation was proposed by Shreeves et al.^22^ GBR-NMF further decomposed the non-negative data matrix *X* into *W*, *A*, and *S* such that

X≈WAS<label>(3)</label>

where *X* is decomposed into an *n* × *q* score matrix *W*, a *q* × *q* auxiliary matrix *A*, and a *q* × *p* matrix *S* containing the (partially) constrained factors. The *S* matrix is partially constrained as the Raman biochemical library. GBR-NMF then updates sequentially the portions of each matrix of the decomposition not assumed to be constrained; for example, the biochemical bases in the matrix *S* are not updated during the optimization.

#### Random Forest and Variable Importance

After conducting the GBR-NMF decomposition on the Raman cellular data, the decomposed chemical scores were classified by random forest (RF) based on the molecular histotype information of the cellular data. RF is a classification method first published by Breiman.^24^ RF contains a collection of decision tree classifiers, wherein each tree generates an output of the classification result from the input data. The forest chooses the final classification result based on the most popular vote over all the trees in the forest.

Variable importance is a measure used to rank the importance of variables during the RF classification.^45^ The out-of-bag (OOB) strategy is commonly used to determine the variable importance.^45–47^ When building each tree, the bootstrap observations which are not used to construct a tree are saved as out-of-bag (OOB) observations. The prediction error on the out-of-bag portion for each tree of the data is recorded as the OOB error. Each predictor variable (e.g., biochemical basis) will later be permuted to calculate the importance of variables. The mean decrease in accuracy (MDA) is calculated by averaging the total difference of OOB errors before and after the random permutation of a single variable over all the trees.

### Workflow of GBR-NMF-RF Framework

**Figure 1.**
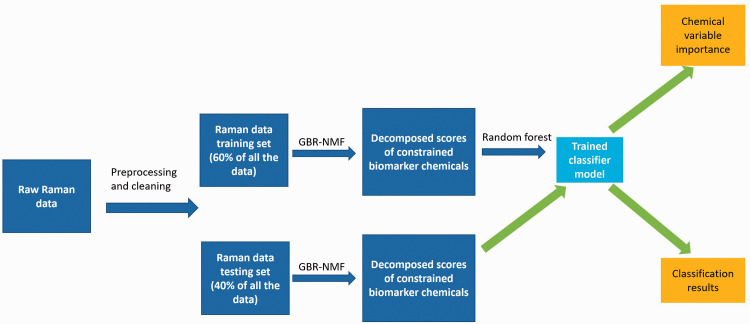
GBR-NMF-RF data analytical framework workflow. After the cellular spectra were processed, the data set was split into 10 training and testing sets pairs. GBR-NMF dimension reduction was performed individually on the training set. The GBR-NMF decomposed chemical scores from the training set were used to random forests (RFs) based on the molecular histotype information of the cellular data. The GBR-NMF decomposed chemical scores from the testing set were used to get the classification performance and variable importance.

A workflow of the GBR-NMF-RF is summarized in Fig. 1. Before performing dimension reduction with GBR-NMF, the RS data set was randomly split into 10 training and testing sets to imitate the case where the model was first trained with prior-knowledge Raman data and then was used to classify new data. Due to the limited number of cells in some categories (e.g., cancer versus non-cancer), 40% of all the data were randomly selected as the testing set each time to make sure both training and testing sets have enough samples from each classification category. A summary of the classification tests and sizes of training and testing sets is presented in Table S3.

After the spectra were pre-processed, GBR-NMF was performed for dimension reduction. The matrix *S* in the GBR-NMF was constrained as the reference chemical RS libraries. One unconstrained factor is allowed in the matrix *S* to represent all the other biochemical change residuals unspecified in the Raman biochemical library. The GBR-NMF algorithm was implemented based on Shreeves et al*.* and available on Github.^22,48^ The group constraining option was omitted in the current work since the underlying groups of the data set are not investigated through GBR-NMF. GBR-NMF was performed on the training set and testing set individually while using the same unconstrained basis matrix obtained from the training set decomposition.

After the dimension reduction step, RF was used to classify the molecular histotypes, and cell line types on the GBR-NMF decomposed biochemical scores. RF was performed with the randomForest package in R.^45,49^ The number of trees specified was 1000 as default. The biomarker contribution results were obtained from the MDA variable importance function of the package. The decomposed biochemical scores from the training set were used to train the RF classifier, and the decomposed scores of chemical bases from the testing set were used to test the performance of the classifier. Every classification test was repeated 10 times to verify the stability of the model as the training and testing set of the data were selected randomly. Relevant metrics such as averaged accuracy, sensitivity, specificity, and variable importance were calculated based on the 10 repeated trials of the randomly split training and testing sets.

We also performed unsupervised NMF to compare with GBR-NMF. A standard NMF function from the scikit-learn package in Python was used to perform unsupervised NMF for comparison.^50^

## Results and Discussion

### Comparison Between Unsupervised NMF and GBR-NMF Chemical Scores

In the current study, Raman spectra were acquired on five cell lines (MCF10A, MCF-7, MDA-MB-231, BT-474, and SK-BR-3) spanning a range of immunochemical features (Table I) over four doses of radiation (0, 10, 30, and 50 Gy) and three harvesting times (18, 42, and 66 h). The GBR-NMF-RF is implemented to analyze the cellular spectra to provide information on biomolecular dynamics while tracking the RT response profiles of molecular histotypes and subtypes. Two RS reference chemical libraries (Tables S1 and S2, and Fig. S1) were used as the constrained chemicals in the GBR-NMF model.

**Figure 2.**
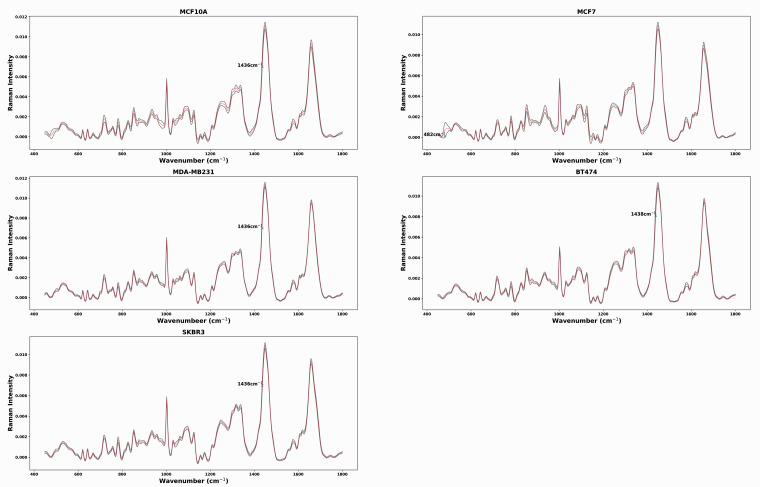
Average Raman spectra obtained from MCF10A, MCF7, MDA-MB-231, BT474, and SKBR3 cells. Shadow spectrum represents ± 1 standard deviation at each wavenumber. For MCAF10A, MDA-MB-231 and SKBR3, the greatest standard deviation occurs at 1436 cm^−1^, which can be attributed to lipids/proteins (CH_2_ scissoring).^30,31,51^ The greatest standard deviation appears at 1438 cm^−1^ for BT474, which can be attributed to the change in saturated fatty acids and triglycerides.^32^ For MCF7, the greatest standard deviation of MCF7 spectra is at 482 cm^−1^ and attributed to glycogen.^52^

The average spectrum (red spectrum) with ±1 standard deviation (shadow spectrum) for each cell line across doses and days is presented in Fig. 2. For MCF10A, MDA-MB-231, and SK-BR-3, the most outstanding standard deviation occurs at 1436 cm^−1^, which can be attributed to lipids/proteins (CH_2_ scissoring).^30,31,51^ The greatest standard deviation appears at 1438 cm^−1^ for BT-474, which can be attributed to the change in saturated fatty acids and triglycerides.^32^ The greatest standard deviation of MCF-7 spectra is at 482 cm^−1^ and attributed to glycogen.^14,23,52^

**Figure 3.**
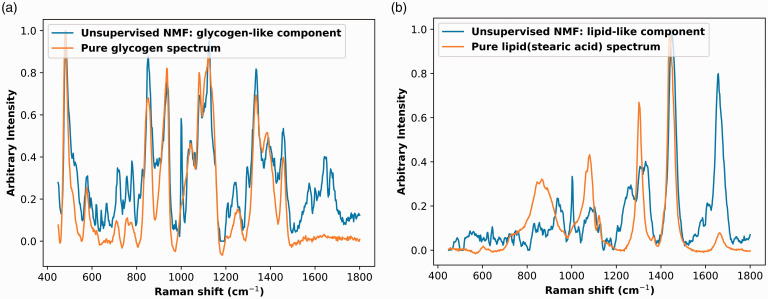
Identifiable bases decomposed by unsupervised NMF overlaid on Raman spectra of pure biochemical. (a) Glycogen-like basis and glycogen. (b) Lipid-like basis and lipid (stearic acid).

Only two chemicals can be identified from the unsupervised NMF decomposed chemical bases, as shown in Fig. 3. These two bases decomposed by unsupervised NMF were similar to two previously identified biochemicals, glycogen, and lipid.^13,14,16^ Other than the first two bases shown in Fig. 3, the remaining decomposed bases were difficult to identify.

**Figure 4.**
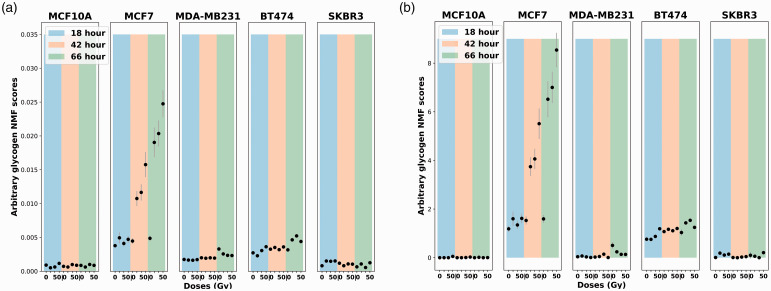
Glycogen score plots comparison between unsupervised NMF and GBR-NMF across five cell lines (MCF10A, MCF7, MDA-MB-231, BT474, and SKBR3). The color bars in the background highlight the harvesting time of sample post-irradiation, 18 h (blue), 42 h (orange), and 66 h (green). Error bars represent ±1 standard error of the glycogen scores. (a) Unsupervised NMF glycogen scores. (b) GBR-NMF glycogen scores (*x*-axis: radiation doses in 0, 10 30, and 50 Gy; *y*-axis: arbitrary glycogen scores generated by GBR-NMF).

To compare the performance of the unsupervised NMF and GBR-NMF, the score plots of glycogen extracted from these two methods are presented in Fig. 4. The plots are displayed over different dose levels (0, 10, 30, and 50 Gy) and time post-irradiation (18, 42, and 66 h). The glycogen variation trend in the score plots was almost identical for GBR-NMF and the unsupervised NMF.

An elevated glycogen level in Fig. 4 was observed in MCF-7 and BT-474 cell lines, which belong to ER-positive and PR-positive molecular histotypes. Meksiarun et al*.* has reported the same observation analyzed with PCA.^12^ Upregulated glycogen levels can be linked to impaired inhibition of glycogen synthase kinase-3 (GSK-3) which regulates glycogen synthesis.^53^ GSK-3 has been reported to have a vital role in regulating the transcription factors of hormone receptors (e.g., estrogen and progesterone).^54,55^

### Classification Performance on Molecular Histotypes, Cancer Versus Non-Cancer Cell Types and Cell Lines

The GBR-NMF-RF analytical framework was tested to classify molecular histotypes, cancer versus non-cancer cell types, and cell lines based on the GBR-NMF decomposed biomarker scores with 10 trials (Tables II to IV). The accuracy was high (>97%) for both 16-chemical and 20-chemical libraries, although the 20-chemical library had a slightly higher accuracy. In Table I, PR and ER histotypes have identical distributions among the cell lines. MCF-7 and BR-474 are PR-positive and ER-positive. MCF10A, MDA-MB-231, and SK-BR-3 are PR-negative and ER-negative. Therefore, ER and PR were combined into one classification test.

**Table II.** Molecular histotypes and cancer versus non-cancer classification results for the 16-chemical library.

	Accuracy (%)	Sensitivity (%)	Specificity (%)
Types	Mean (min;max)	Mean (min;max)	Mean (min;max)
HER2	98.3 (97.7; 98.9)	98.5 (97.2; 99.6)	98.5 (97.2; 99.6)
PR and ER	98.8 (98.1; 99.4)	98.9 (97.9; 99.7)	98.9 (97.9; 99.7)
Ki67	99.4 (98.5; 100.)	99.6 (98.9; 100.)	99.6 (98.9; 100.)
Cancer or not	99.5 (99.2; 100.)	97.5 (95.7; 97.5)	97.5 (95.7; 97.5)

**Table III.** Molecular histotypes and cancer versus non-cancer classification results for the 20-chemical library.

	Accuracy (%)	Sensitivity (%)	Specificity (%)
Types	Mean (min;max)	Mean (min;max)	Mean (min;max)
HER2	98.9 (97.9; 99.1)	98.8 (97.9; 99.7)	98.8 (97.9; 99.7)
PR and ER	99.4 (98.9; 99.8)	99.9 (99.6; 100.)	99.9 (99.6; 100.)
Ki67	99.7 (99.2; 100.)	98.0 (96.2; 99.2)	98.0 (96.2; 99.2)
Cancer or not	99.7 (99.3; 100.)	98.9 (97.1; 100.)	98.9 (97.1; 100.)

**Table IV.** Cell type validation results for the two reference chemical libraries.

16-chemical library accuracy (%)	20-chemical library accuracy (%)
Mean (min;max)	Mean (min;max)
98.0 (96.2; 99.2)	98.5 (97.5; 99.4)

Overall, the framework had an outstanding performance in each of the molecular histotype and cancer-or-not classification tests resulting in high accuracy (>97.0%), high sensitivity (>97.0%), and high specificity (>97.0%) for both the 16-chemical (Table II) and 20-chemical libraries (Table III). The 20-chemical library strategy slightly outperforms the 16-chemical library except forKi67 classification. When comparing the results of 16-chemical and 20-chemical reference libraries, the differences across different metrics were within 2% of the means.

The framework also performed well in the multi-class classification of cell line types, yielding high accuracy (>98.0%) for both the 16-chemical and 20-chemical libraries (Table IV). Again, the 20-chemical library classification average accuracy (98.5%) is slightly improved over the result of the 16-chemical library (98.0%).

### Variable Importance of Chemicals and High Lipids Contribution to Histotype Classification

**Figure 5.**
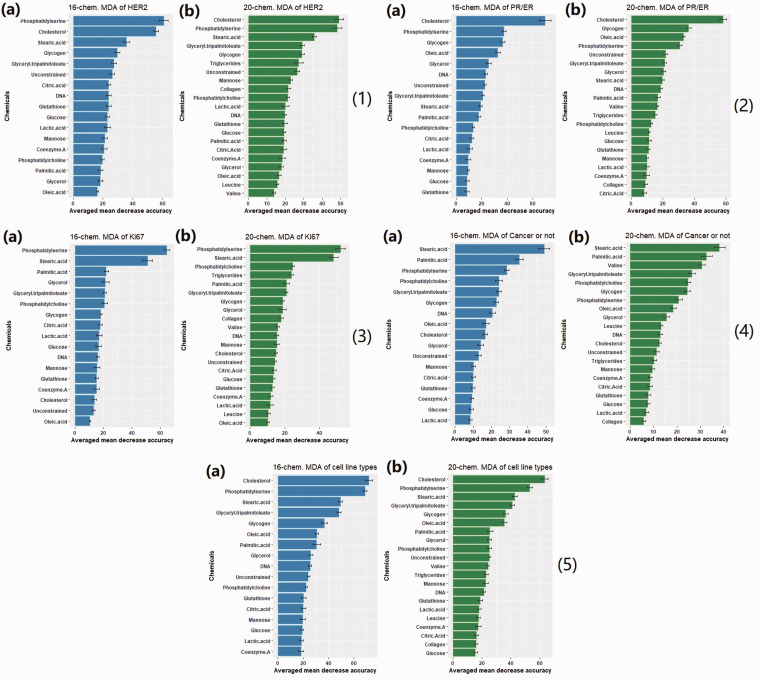
Variable importance plots of the classification tests with Random forest. (a) Constrained with 16 chemicals plus one unconstrained basis. (b) Constrained with 20 chemicals plus one unconstrained basis (1) HER2 (2) PR/ER (3) Ki67 (4) Cancer or not (5) Cell line type. Glycogen and lipids (cholesterol, stearic acid, palmitic acid, oleic acid, and glyceryl triplamitoleate) are often ranked high across different classification tests.

The variable importance plots (i.e., biochemical bases) of 10 trials for each classification test are presented in Fig. 5. The *y*-axis is the reference chemicals tested in the GBR-NMF-RF analysis framework, and *x*-axis is the averaged mean decrease accuracy of 10 trials. The variables with larger values of MDA are ranked higher in terms of their importance in the classification. In Fig. 5, different types of lipids including cholesterol, fatty acids (stearic acid, oleic acid, and palmitic acid), phospholipids (phosphatidylserine and phosphatidylcholine), and triglycerides (glyceryl tripalmitoleate and triglycerides mix) were commonly ranked high (top 5) across all the molecular histotype classification tests. Glycogen also demonstrated high importance in HER2, PR/ER, and cancer-or-not classification tests. Valine, an amino acid, was only regarded as important to distinguish cell lines as cancer or non-cancer.

**Figure 6.**
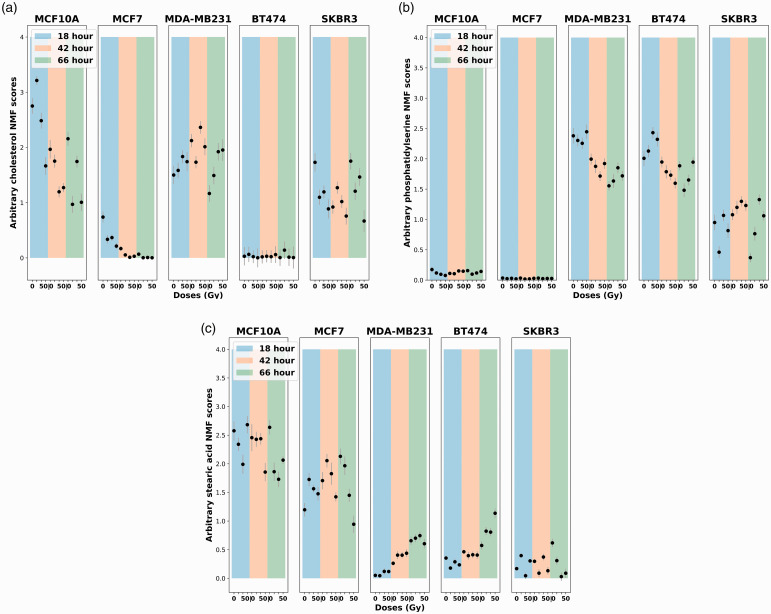
Lipids score plots across five cell lines (MCF10A, MCF7, MDA-MB-231, BT474, and SKBR3). The color bars in the background highlight the harvesting time of sample post-irradiation, 18 h (blue), 42 h (orange), and 66 h (green). Error bars represent ±1 standard error of the glycogen scores. (a) Cholesterol scores. (b) Phosphatidylserine scores (c) Stearic acid scores (*x*-axis: radiation doses in 0, 10 30, and 50 Gy; *y*-axis: arbitrary lipids scores generated using GBR-NMF).

Although various lipids were observed as high-contributing chemicals, cholesterol, phosphatidylserine, and stearic acid stood out as the top three chemicals in multiple classifications (HER2, Ki67, and cell line types) in Fig. 5. The GBR-NMF decomposed score plots of cholesterol, phosphatidylserine, and stearic acid are displayed in Fig. 6. The plots are displayed over different dose levels (0, 10, 30, and 50 Gy) and time post-irradiation (18, 42, and 66 h).

Cholesterol is ranked as the most important chemical in the PR/ER classification test in Fig. 5(2). The three PR/ER-negative cell lines (MCF10A, MDA-MB-231, and SK-BR-3) also exhibited high levels of cholesterol content in Fig. 6a. Cholesterol has been directly linked to the implication of the development of estrogen receptor-positive breast cancer via the metabolite 27-hydroxycholesterol.^56–59^ 27-hydroxycholesterol may act as a modulator in the cholesterol metabolism.^60,61^

High levels of phosphatidylserine were presented in Ki67-high cell lines (MDA-MB-231, BT-474, and SK-BR-3) in Fig. 6b. The Ki67-low cell lines (MCF10A and MCF-7) have high stearic acid scores in Fig. 6c. Ki67 has been widely acknowledged as a proliferation marker for human tumor cells.^62^ Phosphatidylserine was found to increase significantly on the surface of tumour cells and facilitate tumour growth and metastasis in the tumor microenvironment.^63,64^ Studies on stearic acid have discovered its role in inhibiting human breast cancer cell proliferation in vitro*.*^65–68^ Our observations of high variable importance of phosphatidylserine and stearic acid in the Ki67 classification test (Fig. 5^2^), high phosphatidylserine scores in Ki67-high cell lines, and high stearic acid scores in Ki67-low cell lines are congruent with previous studies on phosphatidylserine and stearic acid metabolism in cancer.

## General Discussion

From the results presented above, RS combined with the GBR-NMF-RF analytical framework exhibited an outstanding ability to classify breast cell lines into molecular histotypes even after treated by RT. The combination of RS and GBR-NMF-RF provides an efficient approach to track the biochemical profiles of different molecular histotypes throughout radiation therapy treatment. The accuracy, sensitivity, and specificity results across all the molecular histotype classifications were high (>97%) under our novel analytical framework. The GBR-NMF-RF analytical framework produced robust molecular histotype classification results across different radiation doses (0, 10, 30, and 50 Gy) in this breast cell data set. Using the GBR-NMF-RF framework along with RS provides the potential to track molecular histotypes under varied conditions in different radiotherapy plans and has the potential to open up new treatment pathways which could be exploited in order to both improve and personalize RT.

The chemicals selected in the reference library were to cover a complete set of molecular compounds based on the cell composition and chemicals involved in cancer metabolism. The original motivation to include two Raman libraries with different sizes was to examine whether the more extensive 20-chemical library would improve the margins of classification accuracy. Although the 20-chemical library did yield slightly higher accuracy, the difference was marginal (<2%) and demonstrates that the 16-chemical library selected captured a vast majority of biological information for the current molecular histotype classification purpose since the differences on the metrics were often less than 2% (in Tables II to IV).

The linkage between Raman biomarkers (e.g., glycogen and lipids (cholesterol, phosphatidylserine, and stearic acid)) and molecular histotypes (PR/ER and Ki67) were uncovered by GBR-NMF generated score plots and variable importance calculated by RF (Figs. 5 and 6). The importance of glycogen and lipids in radiation response and cancer metabolism has been previously reported.^12–16,23^ The insights on how glycogen and lipids (cholesterol, phosphatidylserine, and stearic acid) can be linked to PR/ER and Ki67 molecular histotypes and how this relates to RT are reported by RS for the first time to our knowledge, although these metabolites have been studied intensively in the cancer research community.^56–59,63–68^

The motivation to add extra chemicals such as leucine and valine into the reference chemical library was to potentially investigate whether RS and the GBR-NMF-RF framework could track changes in protein or amino acids in the breast cell lines. Proteins are difficult to trace because the amino acid composition of proteins can vary greatly.^69^ Valine and leucine were selected given their emerging importance in cancer biology.^42^ Understanding the requirements for valine, leucine, and other amino acids in the TCA may open up potential ways to target their utilization in combination with radiation therapy.^70^ In this study, valine only showed a high contribution to cancer or non-cancer classification. Although tracking complex proteins with RS is currently challenging, possible improvements can still be achieved with advanced data analysis methods to extract relevant information.

Using RS with the novel GBR-NMF-RF analytical framework, the interpretability of the underlying biological information in our RS cellular data set was significantly improved over the previous studies.^33,13–16,23^ Combining RS and GBR-NMF-RF demonstrates a great potential to track the molecular histotypes and therefore monitor biochemical dynamics in specific tumour environments during radiation therapy treatments. Although a range of breast cancer cell lines were covered in the current study, future studies on other types of breast cancer cell lines or non-breast cancer cell lines should be conducted. Validation of the current results obtained using RS using other techniques such as mass spectrometry is also a promising direction to pursue for future research.

## Conclusion

The GBR-NMF-RF analytical framework is capable of monitoring known immunohistochemical profiles of different breast cancer cell lines using Raman spectra collected subsequent to treatment with varying dose levels of ionising radiation. The GBR-NMF-RF framework had classification results for immunohistochemical profiles with high accuracy (>97%), high sensitivity (>97%), and high specificity (>97%). The variable importance function from the random forest also provided information about the high contributions of RS identified biomarkers (e.g., glycogen and lipids (cholesterol, phosphatidylserine, and stearic acid)) in molecular histotype classification tests and how those markers relate to RT. The combination of RS and GBR-NMF-RF gives a robust technique to track the molecular histotypes throughout the radiation therapy treatment.

## Supplemental Material

sj-pdf-1-asp-10.1177_00037028211035398 - Supplemental material for Group and Basis Restricted Non-Negative Matrix Factorization and Random Forest for Molecular Histotype Classification and Raman Biomarker Monitoring in Breast Cancer Click here for additional data file. Supplemental material, sj-pdf-1-asp-10.1177_00037028211035398 for Group and Basis Restricted Non-Negative Matrix Factorization and Random Forest for Molecular Histotype Classification and Raman Biomarker Monitoring in Breast Cancer by Xinchen Deng, Kirsty Milligan, Ramie Ali-Adeeb, Phillip Shreeves, Alexandre Brolo, Julian J. Lum, Jeffrey L. Andrews and Andrew Jirasek in Applied Spectroscopy

## References

[1] Baskar R. Lee K.A. Yeo R. Yeoh K.W. “Cancer and Radiation Therapy: Current Advances and Future Directions”. Int. J. Med. Sci. 2012; 9(3): 193–199. doi: 10.7150/ijms.3635. 22408567 10.7150/ijms.3635 PMC3298009

[2] Ghaye B. Wanet M. El Hajjam M. “Imaging After Radiation Therapy of Thoracic Tumours”. Diagn. Interv. Imaging. 2016; 97(10): 1037–1052. doi:10.1016/j.diii.2016.06.019. 27567554 10.1016/j.diii.2016.06.019

[3] Riyahi S. Choi W. Liu C.J. Zhong H. , et al. “Quantifying Local Tumour Morphological Changes with Jacobian Map for Prediction of Pathologic Tumour Response to Chemo-Radiotherapy in Locally Advanced Esophageal Cancer”. Phys. Med. Biol. 2018; 63(14): 145020 doi:10.1088/1361-6560/aacd22. 29911659 10.1088/1361-6560/aacd22 PMC6064042

[4] J.M. Moran, M.A. Elshaikh, T.S. Lawrence. “Radiotherapy: What Can Be Achieved By Technical Improvements in Dose Delivery?” Lancet Oncol. 2005. 6(1): 51–58. doi:10.1016/S1470-2045(04)01713-9. 10.1016/S1470-2045(04)01713-9 15629276

[5] Tang L. Wei F. Wu Y. He Y. , et al. “Role of Metabolism in Cancer Cell Radioresistance and Radiosensitization Methods”. J. Exp. Clin. Cancer Res. 2018; 37(1): 87 doi:10.1186/s13046-018-0758-7. 29688867 10.1186/s13046-018-0758-7 PMC5914062

[6] Zaha D.C. “Significance of Immunohistochemistry in Breast Cancer”. World J. Clin. Onco. 2014; 5(3): 382–392. doi:10.5306/wjco.v5.i3.382. 10.5306/wjco.v5.i3.382 PMC4127609 25114853

[7] Vuong D.S. Peter T. Green B. Cummings M.C. , et al. “Molecular Classification of Breast Cancer”. Virchows Archiv. 2014; 465(1): 1–14. doi: 10.1007/s00428-014-1593-7. 24878755 10.1007/s00428-014-1593-7

[8] Livasy C.A. Perou C.M. Karaca G. Cowan D.W. , et al. “Identification of a Basal-Like Subtype of Breast Ductal Carcinoma In Situ”. Hum Pathol. 2007; 38(2): 197–204. doi: 10.1016/j.humpath.2006.08.017. 17234468 10.1016/j.humpath.2006.08.017

[9] Valla M. Vatten L.J. Engstrøm M.J. Haugen O.A. , et al. “Molecular Subtypes of Breast Cancer: Long-term Incidence Trends and Prognostic Differences”. Cancer Epidemiol. Biomark. Prev. 2016; 25(12): 1625–1634. doi: 10.1158/1055-9965.EPI-16-0427. 10.1158/1055-9965.EPI-16-0427 27672056

[10] Aristei C. Perrucci E. Alì E. Marazzi F. , et al. “Personalization in Modern Radiation Oncology: Methods, Results and Pitfalls. Personalized Interventions and Breast Cancer”. Front Oncol. 2021; 11: 616042 doi: 10.3389/fonc.2021.616042. 33816246 10.3389/fonc.2021.616042 PMC8012886

[11] Bibault J.E. Fumagalli I. Ferté C. Chargari C. , et al. “Personalized Radiation Therapy and Biomarker-Driven Treatment Strategies: A Systematic Review”. Cancer Metastas. Rev. 2013; 32(3-4): 479–492. doi: 10.1007/s10555-013-9419-7. 10.1007/s10555-013-9419-7 23595306

[12] Meksiarun P. Aoki P.H.B. Nest S.J.V. Sobral-Filho R.G. , et al. “Breast Cancer Subtype Specific Biochemical Responses to Radiation”. Analyst. 2018; 143(16): 3850–3858. doi:10.1039/C8AN00345A. 30004539 10.1039/c8an00345a

[13] Matthews Q. Jirasek A. Lum J. Brolo A. “Biochemical Signatures of In Vitro Radiation Response in Human Lung, Breast and Prostate Tumour Cells Observed with Raman Spectroscopy”. Phys. Med. Biol. 2011; 56: 6839–6855. doi:10.1088/0031-9155/56/21/006. 21971286 10.1088/0031-9155/56/21/006

[14] Matthews Q. Isabelle M. Harder S.J. Smazynski J. , et al. “Radiation-Induced Glycogen Accumulation Detected by Single Cell Raman Spectroscopy Is Associated with Radioresistance that Can Be Reversed by Metformin”. PLoS One. 2015; 10(8): e0135356 doi:10.1371/journal.pone.0135356. 26280348 10.1371/journal.pone.0135356 PMC4539228

[15] Harder S.J. Isabelle M. DeVorkin L. Smazynski J. , et al. “Raman Spectroscopy Identifies Radiation Response in Human Non-Small Cell Lung Cancer Xenografts”. Sci. Rep. 2016; 6: 21006 doi:10.1038/srep21006. 26883914 10.1038/srep21006 PMC4756358

[16] Deng X. Ali-Adeeb R. Andrews J.L. Shreeves P. , et al. “Monitor Ionizing Radiation-Induced Cellular Responses with Raman Spectroscopy, Non-Negative Matrix Factorization, and Non-Negative Least Squares”. Appl. Spectrosc. 2020; 74(6): 701–711. doi:10.1177/0003702820906221. 32098482 10.1177/0003702820906221

[17] Paidi S.K. Monterroso Diaz P. Dadgar S. Jenkins S.V. , et al. “Label-Free Raman Spectroscopy Reveals Signatures of Radiation Resistance in the Tumor Microenvironment”. Cancer Res. 2019; 79(8): 2054–2064. doi:10.1158/0008-5472.CAN-18-2732. 30819665 10.1158/0008-5472.CAN-18-2732 PMC6467810

[18] Feng X. Moy A.J. Nguyen H.T.M. Zhang J. , et al. “Raman Active Components of Skin Cancer”. Biomed. Opt. Exp. 2017; 8(6): 2835–2850. doi:10.1364/BOE.8.002835. 10.1364/BOE.8.002835 PMC5480433 28663910

[19] D.D. Lee, H.S. Seung. “Algorithms for Non-negative Matrix Factorization”. In: Proceedings of the 13th International Conference on Neural Information Processing Systems (NIPS '00). Denver, Colorado; 1 Jan 2000. Pp. 535--541.

[20] Sajda P. Du S. Parra L. “Recovery of Constituent Spectra Using Non-Negative Matrix Factorization. Proc. SPIE. 2003; 5207: 321–331. doi:10.1117/12.504676.

[21] Li H. Adal T. Wang W. Emge D. , et al. “Non-Negative Matrix Factorization with Orthogonality Constraints and its Application to Raman Spectroscopy”. J. VLSI Signal Process. Syst. Signal Image Video Technol. 2007; 48(1-2): 83–97. doi:10.1007/s11265-006-0039-0.

[22] P. Shreeves, J.L. Andrews, X. Deng, R. Ali-Adeeb, et al. “Nonnegative Matrix Factorization with Group and Basis Restrictions”. ArXiv. 2021. https://arxiv.org/abs/2107.00744.

[23] Milligan K. Deng X. Shreeves P. Ali-Adeeb R. , et al. “Raman Spectroscopy and Group and Basis-Restricted Non Negative Matrix Factorisation Identifies Radiation Induced Metabolic Changes in Human Cancer Cells”. Sci. Rep. 2021; 11(1): 3853 doi:10.1038/s41598-021-83343-5. 33594122 10.1038/s41598-021-83343-5 PMC7886912

[24] Breiman L. “Random Forests”. Mach. Learn. 2001; 45(1): 5–32. doi:10.1023/A:1010933404324.

[25] Prat A. Pineda E. Adamo B. Galván P. , et al. “Clinical Implications of the Intrinsic Molecular Subtypes of Breast Cancer. Breast. 2015; 24(Suppl 2): S26–35. doi:10.1016/j.breast.2015.07.008. 26253814 10.1016/j.breast.2015.07.008

[26] Dai X. Li T. Bai Z. Yang Y. , et al. “Breast Cancer Intrinsic Subtype Classification, Clinical Use and Future Trends”. Am. J. Cancer Res. 2015; 5(10): 2929–2943. 26693050 PMC4656721

[27] Li L.T. Jiang G. Chen Q. Zheng J.N. “Ki67 is a Promising Molecular Target in the Diagnosis of Cancer (Review)”. Mol. Med. Rep. 2015; 11(3): 1566–1572. doi:10.3892/mmr.2014.2914. 25384676 10.3892/mmr.2014.2914

[28] Abubakar M. Figueroa J. Ali H.R. Blows F. , et al. “Combined Quantitative Measures of ER, PR, HER2, and KI67 Provide More Prognostic Information than Categorical Combinations in Luminal Breast Cancer”. Mod. Pathol. 2019; 32(9): 1244–1256. doi:10.1038/s41379-019-0270-4. 30976105 10.1038/s41379-019-0270-4 PMC6731159

[29] G.M. Cooper. “The Molecular Composition of Cells”. The Cell: A Molecular Approach. Washington, DC: ASM Press; Sunderland, Massachussetts: Sinauer Associates, 2000. Chap. 2. https://www.ncbi.nlm.nih.gov/books/NBK9839/ [accessed Jul 28 2021].

[30] De Gelder J. De Gussem K. Vandenabeele P. Moens L. “Reference Database of Raman Spectra of Biological Molecules”. J. Raman Spectrosc. 2007; 38(9): 1133–1147. doi:10.1002/jrs.1734.

[31] Talari A.C.S. Movasaghi Z. Rehman S. Rehman I. “Raman Spectroscopy of Biological Tissues”. Appl. Spectrosc. Rev. 2015; 50(1): 46–111. doi:10.1080/05704928.2014.923902.

[32] Czamara K. Majzner K. Pacia M.Z. Kochan K. , et al. “Raman Spectroscopy of Lipids: A Review”. J. Raman Spectrosc. 2015; 46(1): 4–20. doi:10.1002/jrs.4607.

[33] Matthews Q. Jirasek A. Lum J. Duan X. , et al. “Variability in Raman Spectra of single Human Tumor Cells Cultured In Vitro: Correlation with Cell Cycle and Culture Confluency”. Appl. Spectrosc. 2010; 64(8): 871–877. doi: 10.1366/000370210792080966. 20719050 10.1366/000370210792080966

[34] Faubert B. Li K.Y. Cai L. Hensley C.T. , et al. “Lactate Metabolism in Human Lung Tumors”. Cell. 2017; 171(2): 358–371.e9. doi:10.1016/j.cell.2017.09.019. 28985563 10.1016/j.cell.2017.09.019 PMC5684706

[35] Hui S. Ghergurovich J. Morscher R. Jang C. , et al. “Glucose Feeds the TCA Cycle Via Circulating Lactate”. Nature. 2017; 551: 115–118. doi:10.1038/nature24057. 29045397 10.1038/nature24057 PMC5898814

[36] S. Zhang, M.W. Hulver, R.P. McMillan, M.A. Cline, et al. “The Pivotal Role of Pyruvate Dehydrogenase Kinases in Metabolic Flexibility”. Nutr. Metab. 2014. 11(10). doi:10.1186/1743-7075-11-10. 10.1186/1743-7075-11-10 PMC3925357 24520982

[37] Borrego-Soto G. Ortiz-López R. Rojas-Martínez A. “Ionizing Radiation-Induced DNA Injury and Damage Detection in Patients with Breast Cancer”. Genet. Mol. Biol. 2015; 38(4): 420–432. doi:10.1590/S1415-475738420150019. 26692152 10.1590/S1415-475738420150019 PMC4763322

[38] Bansal A. Simon M.C. “Glutathione Metabolism in Cancer Progression and Treatment Resistance”. J. Cell. Biol. 2018; 217(7): 2291–2298. doi:10.1083/jcb.201804161. 29915025 10.1083/jcb.201804161 PMC6028537

[39] Kennedy L. Sandhu J.K. Harper M.E. Cuperlovic-Culf M. “Role of Glutathione in Cancer: From Mechanisms to Therapies”. Biomolecules. 2020; 10(10): 1429 doi:10.3390/biom10101429. 10.3390/biom10101429 PMC7600400 33050144

[40] Reily C. Stewart T.J. Renfrow M.B. Novak J. , et al. “Glycosylation in Health and Disease”. Nat. Rev. Nephrol. 2019; 15: 346–366. doi:10.1038/s41581-019-0129-4. 30858582 10.1038/s41581-019-0129-4 PMC6590709

[41] Ferreira I.G. Pucci M. Venturi G. Malagolini N. , et al. “Glycosylation as a Main Regulator of Growth and Death Factor Receptors Signaling”. Int. J. Mol. Sci. 2018; 19(2): 580 doi:10.3390/ijms19020580. 10.3390/ijms19020580 PMC5855802 29462882

[42] Nguyen D.M. “Molecular Heterogeneity of Inflammatory Breast Cancer: A Hyperproliferative Phenotype”. Clin. Cancer Res. 2006; 12(17): 5047–5054. doi:10.1158/1078-0432.CCR-05-2248. 16951220 10.1158/1078-0432.CCR-05-2248

[43] Lieu E.L. Nguyen T. Rhyne S. Kim J. “Amino Acids in Cancer”. Exp. Mol. Med. 2020; 52(1): 15–30. doi:10.1038/s12276-020-0375-3. 31980738 10.1038/s12276-020-0375-3 PMC7000687

[44] Lodish H, Berk A, Zipursky SL, Matsudaira P, et al. “Collagen: The Fibrous Proteins of the Matrix”. In: H.F. Lodish, A. Berk, S.L. Zipersky, et al., editors. Molecular Cell Biology. New York: W.H. Freeman, 2000. Section 22.3. www.ncbi.nlm.nih.gov/books/NBK21582/ [accessed Jul 28 2021].

[45] A. Liaw, M. Wiener. “Classification and Regression by randomForest R News”. 2002. 2(3):18–22. https://CRAN.R-project.org/doc/Rnews/ [accessed July 21].

[46] H. Han, X. Guo, H. Yu. “Variable Selection using Mean Decrease Accuracy and Mean Decrease Gini Based on Random Forest”. In: 2016 7th IEEE International Conference on Software Engineering and Service Science (ICSESS). Beijing, China: 26--28 Aug 2016. 219–224. doi: 10.1109/ICSESS.2016.7883053.

[47] Altmann A. Toloşi L. Sander O. Lengauer T. “Permutation Importance: A Corrected Feature Importance Measure”. Bioinformatics. 2010; 26(10): 1340–1347. doi:10.1093/bioinformatics/btq134. 20385727 10.1093/bioinformatics/btq134

[48] J.L. Andrews and P. Shreeves. “Group and Basis Restricted Non-Negative Matrix Factorization”. 2020. https://github.com/its-likeli-jeff/GBRNMF [accessed July 21].

[49] R Core Team. “R: A Language and Environment for Statistical Computing”. 2020. https://www.R-project.org [accessed July 21].

[50] F. Pedregosa, G. Varoquaux, A. Gramfort, V. Michel, et al. “Scikit-Learn: Machine Learning in Python”. J. Mach. Learn. Res. 2011. 12(85): 2825--2830.

[51] Surmacki J.M. Woodhams B.J. Haslehurst A. Ponder B.A.J. , et al. “Raman Micro-Spectroscopy for Accurate Identification of Primary Human Bronchial Epithelial Cells”. Sci Rep. 2018; 8: 12604 doi:10.1038/s41598-018-30407-8. 30135442 10.1038/s41598-018-30407-8 PMC6105656

[52] Duraipandian S. Traynor D. Kearney P. Martin C. , et al. “Raman Spectroscopic Detection of High-Grade Cervical Cytology: Using Morphologically Normal Appearing Cells”. Sci Rep. 2018; 8: 15048 doi:10.1038/s41598-018-33417-8. 30301922 10.1038/s41598-018-33417-8 PMC6177468

[53] Miyashita K. Kawakami K. Nakada M. Mai W. , et al. “Potential Therapeutic Effect of Glycogen Synthase Kinase 3 Beta Inhibition against Human Glioblastoma”. Clin. Cancer Res. 2009; 15(3): 887–897. doi:10.1158/1078-0432.CCR-08-0760. 19188159 10.1158/1078-0432.CCR-08-0760

[54] Grisouard J. Medunjanin S. Hermani A. Shukla A. , et al. “Glycogen Synthase Kinase-3 Protects Estrogen Receptor Alpha from Proteasomal Degradation and is Required for Full Transcriptional Activity of the Receptor”. Mol. Endocrinol. 2007; 21(10): 2427–2439. doi: 10.1210/me.2007-0129. 17609434 10.1210/me.2007-0129

[55] Wang S. Li Y. Hsu P.H. Lee S.Y. , et al. “Progesterone Receptor A Stability is Mediated by Glycogen Synthase Kinase-3 Beta in the Brca1-Deficient Mammary Gland. J. Biol. Chem. 2013; 288(36): 26265–26274. doi:10.1074/jbc.M113.476556. 23880761 10.1074/jbc.M113.476556 PMC3764830

[56] Nelson E.R. Wardell S.E. Jasper J.S. Park S. , et al. “27-Hydroxycholesterol Links Hypercholesterolemia and Breast Cancer Pathophysiology”. Science. 2013; 342(6162): 1094–1098. 24288332 10.1126/science.1241908 PMC3899689

[57] Warner M. Gustafsson J. “27-Hydroxycholesterol Links Hypercholesterolemia and Breast Cancer Pathophysiology”. N. Engl. J. Med. 2014; 270(6): 572–573. doi:10.1056/NEJMcibr1315176.

[58] Baek A.E. Nelson E.R. “The Contribution of Cholesterol and Its Metabolites to the Pathophysiology of Breast Cancer”. Horm. Cancer. 2016; 7(4): 219–228. doi:10.1007/s12672-016-0262-5. 27020054 10.1007/s12672-016-0262-5 PMC4930376

[59] Potluri R. Carter P.R. Lavu D. Bainey K.R. “The Interplay Between Cholesterol and Breast Cancer: Is There a Potential Role for Statin Therapy? Future Oncol. 2018; 14(19): 1885–1888. doi:10.2217/fon-2018-0160. 30051723 10.2217/fon-2018-0160

[60] Souidi M. Dubrac S. Parquet M. Milliat F. , et al. “Effects of Dietary 27-Hydroxycholesterol on Cholesterol Metabolism and Bile Acid Biosynthesis in the Hamster”. Can. J. Physiol. Pharmacol. 2003; 81(9): 854–863. doi: 10.1139/y03-079. 14614521 10.1139/y03-079

[61] An Y. Zhang D.D. Yu H.L. Ma W.W. , et al. “27-Hydroxycholesterol Regulates Cholesterol Synthesis and Transport in C6 Glioma Cells”. Neurotoxicology. 2017; 59: 88–97. doi: 10.1016/j.neuro.2017.02.001. 28167099 10.1016/j.neuro.2017.02.001

[62] Sun X. Kaufman P.D. “Ki-67: More than a Proliferation Marker”. Chromosoma. 2018; 127(2): 175–186. doi:10.1007/s00412-018-0659-8. 29322240 10.1007/s00412-018-0659-8 PMC5945335

[63] Birge R. Boeltz S. Kumar S. Carlson J. , et al. “Phosphatidylserine is a Global Immunosuppressive Signal in Efferocytosis, Infectious Disease, and Cancer”. Cell Death Differ. 2016; 23: 962–978. doi:10.1038/cdd.2016.11. 26915293 10.1038/cdd.2016.11 PMC4987730

[64] Chang W. Fa H. Xiao D. Wang J. “Targeting Phosphatidylserine for Cancer therapy: Prospects and Challenges”. Theranostics. 2020; 10(20): 9214–9229. doi:10.7150/thno.45125. 32802188 10.7150/thno.45125 PMC7415799

[65] Wickramasinghe N.S. Jo H. McDonald J.M. Hardy R.W. “Stearate Inhibition of breast Cancer Cell Proliferation. A Mechanism Involving Epidermal Growth Factor Receptor and G-Proteins”. Am. J. Pathol. 1996; 148: 987–995. 8774153 PMC1861711

[66] Hardy R.W. Wickramasinghe N.S. Ke S.C. Wells A. “Fatty Acids and Breast Cancer Cell Proliferation”. Adv. Exp. Med. Biol. 1997; 422: 57–69. 9361815 10.1007/978-1-4757-2670-1_5

[67] Evans L.M. Stephanie L.C. Gene P.S. Robert W.H. “Stearate Preferentially Induces Apoptosis in Human Breast Cancer Cells”. Nutr. Cancer. 2009; 61(5): 746–753. 19838949 10.1080/01635580902825597 PMC2946230

[68] Khan A.A. Alanazi A.M. Jabeen M. Chauhan A. , et al. “Design, Synthesis and In Vitro Anticancer Evaluation of a Stearic Acid-Based Ester Conjugate”. Anticancer Res. 2013; 33(6): 2517–24. 23749903

[69] H. Lodish, A. Berk, S.L. Zipursky, P. Matsudaira, et al. “Hierarchical Structure of Proteins”. In: H.F. Lodish, A. Berk, S.L. Zipersky, et al., editors. Molecular Cell Biology. New York: W.H. Freeman, 2000. Section 3.1. www.ncbi.nlm.nih.gov/books/NBK21581/ [accessed Jul 28 2021].

[70] Anderson N.M. Mucka P. Kern J.G. Feng H. “The Emerging Role and Targetability of the TCA Cycle in Cancer Metabolism”. Protein & Cell. 2018; 9(2): 216–237. doi:10.1007/s13238-017-0451-1. 28748451 10.1007/s13238-017-0451-1 PMC5818369

