# Supplementary material for: Group and Basis Restricted Non-Negative Matrix Factorization and Random Forest for Molecular Histotype Classification and Raman Biomarker Monitoring in Breast Cancer
Source: Appl Spectrosc. 2021 Aug 6;76(4):462–74. doi: 10.1177/00037028211035398 (PMC9003771; doi:10.1177/00037028211035398)
Supplement: sj-pdf-1-asp-10.1177_00037028211035398 - Supplemental material for Group and Basis Restricted Non-Negative Matrix Factorization and Random Forest for Molecular Histotype Classification and Raman Biomarker Monitoring in Breast Cancer [file sj-pdf-1-asp-10.1177_00037028211035398.pdf]

Supplemental Material

Group and Basis Restricted Non-Negative Matrix Factorization and Random Forest for Molecular Histotype Classification and Raman Biomarker Monitoring in Breast Cancer

Xinchen Deng[1], Kirsty Milligan[1], Ramie Ali-Adeeb[1], Phillip Shreeves[2], Alexandre Brolo[3], Julian J. Lum[4,5], Jeffrey L. Andrews[2], and Andrew Jirasek[1]*

Figure S1. Reference Raman spectra of chemical bases.

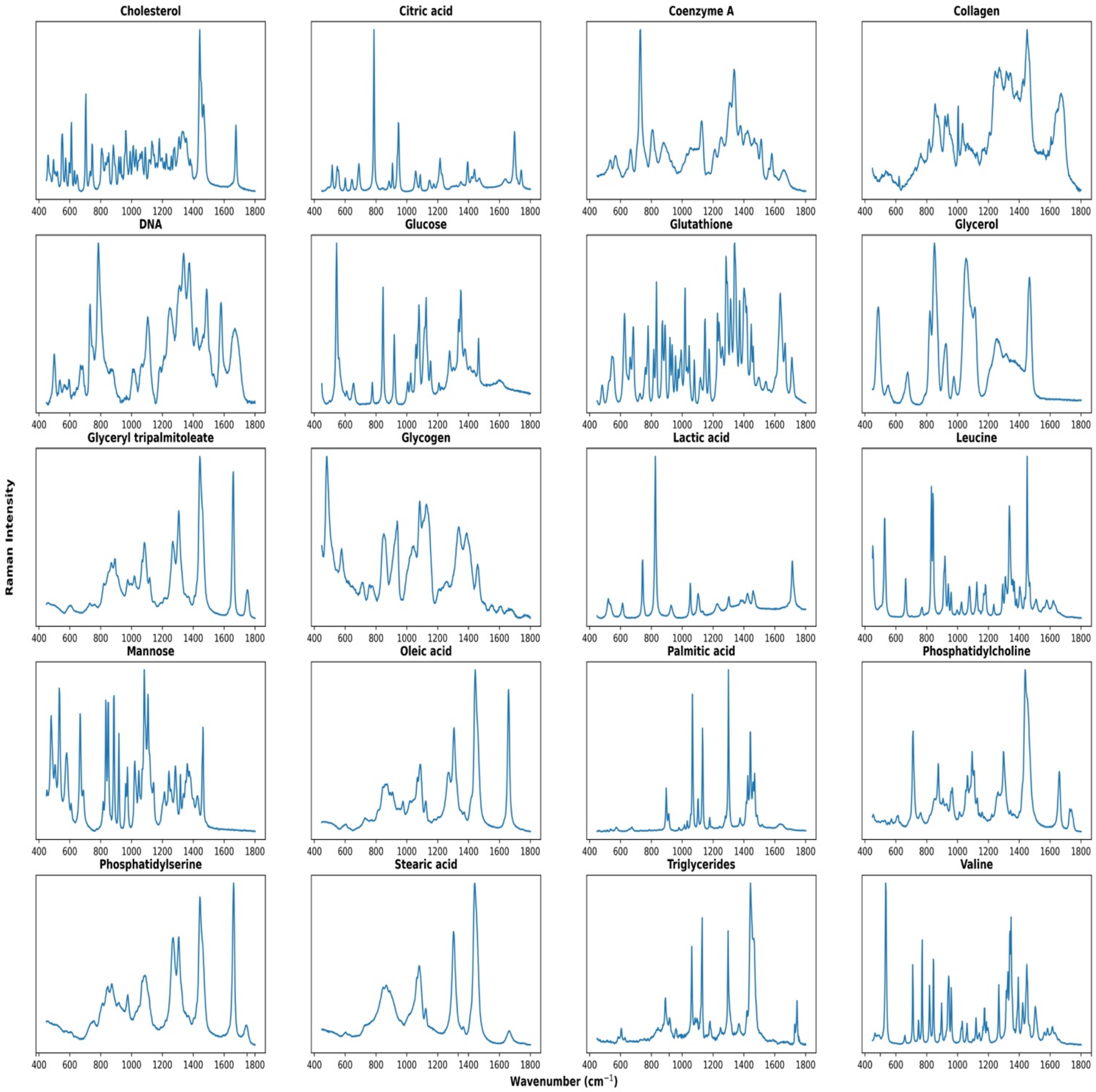

Table S1. The 16-reference biochemical Raman library.

Cholesterol
Citric acid
Coenzyme A
DNA
Glucose
Glutathione
Glycerol
Glyceryl tripalmitoleate
Glycogen
Lactic acid
Mannose
Oleic acid
Palmitic acid
Phosphatidylcholine
Phosphatidylserine
Steric acid

Table S2. The 20-reference biochemical Raman chemical library.

Cholesterol
Citric acid
Coenzyme A
Collagen
DNA
Glucose
Glutathione
Glycerol
Glyceryl tripalmitoleate
Glycogen
Lactic acid
Leucine
Mannose
Oleic acid
Palmitic acid
Phosphatidylcholine
Phosphatidylserine
Stearic acid
Triglycerides
Valine

Table S3. Summary of classification tests and training and testing set sizes.

Classification tests[a]	Positive cell lines	Negative cell lines	Positive training	Negative training	Positive testing	Negative testing
HER2	MCF10A, MCF-7, MDA-MB-231	BT-474, SK-BR-3	432	288	288	192

PR/ER	MCF10A, MDA-MB-231, S-KBR-3	MCF-7, BT-474	432	288	288	192
Ki67	MCF10A, MCF-7	MDA-MB-231, BT-474, SK-BR-3	288	432	192	288
Cancer or not	MCF-7, MDA-MB-231, BT-474, S-KBR-3	MCF10A	576	144	384	96

[a]Cell subtype (MCF10A, MCF-7, MDA-MB-231, BT-474, SK-BR-3) multiclass classification: Each cell line is a class, with 144 training and 96 testing spectra in each class.
